# Circadian Timekeeping Through Nutritional and Metabolic Sensory Networks

**DOI:** 10.3390/nu18071133

**Published:** 2026-03-31

**Authors:** Erin N. Doherty, Lauren N. Woodie

**Affiliations:** Department of Pharmacology and Physiology, The George Washington University School of Medicine and Health Sciences, Washington, DC 20052, USA; erin.doherty@gwu.edu

**Keywords:** circadian rhythms, nutrition, metabolism, sensory

## Abstract

Circadian rhythms are predictable biological patterns that recur about every 24 h and, in mammals such as humans, are entrained to daylight by the hypothalamic suprachiasmatic nucleus (SCN). Although light is a potent zeitgeber for the SCN, cells outside of the SCN can synchronize to daily nutrient and metabolic cues. In these tissues, nutrient metabolic processes are regulated by the molecular clock in anticipation of food availability or scarcity. Furthermore, nutrients and metabolic processes themselves may act upon members of the molecular clock to regulate their expression and activity. These interactions maintain synchrony between the SCN and food-entrainable clocks when activity and nutrient intake align. However, the light-entrainable SCN and food-entrainable clocks can become desynchronized, particularly in modern society where humans are commonly exposed to shift work and jet lag. Therefore, the mechanisms for sensing nutrients at specific times of day are critical components of circadian timekeeping and organismal homeostasis. In the following narrative review, we aim to synthesize current evidence on time-of-day-dependent nutrient sensing in mammalian systems, examine how nutrient-derived signals and metabolic processes interact with molecular clock mechanisms across cellular and tissue levels, and evaluate the integration of central and peripheral clocks in regulating gene expression, energy utilization, and organismal homeostasis, including the impacts of feeding cycles and circadian disruption. While previous reviews have discussed circadian nutrient metabolism, this review provides conceptual support for the role of nutrients as time-of-day signaling mechanisms.

## 1. Introduction

Life in all forms exists on a timescale ranging from years to minutes. One of the most relevant timescales to you as the reader is the daily rhythm of behavior and physiology known as circadian rhythms [1,2]. The word circadian comes from the Latin circa meaning “around” and dies meaning “day” indicating that circadian rhythms are predictable biological patterns that occur every day [3]. This timescale evolved in response to the 24 h it takes for the Earth to rotate on its axis and the ~12 h of sunlight and darkness that surface-dwelling mammals experience [3,4,5,6].

**Figure 1:**
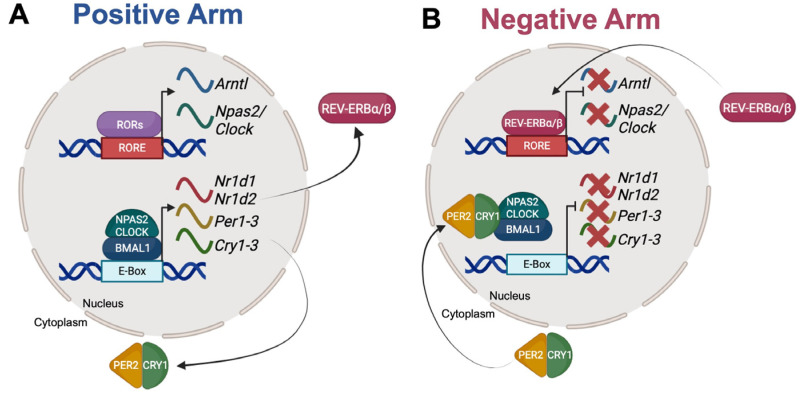
The Mammalian Circadian Clock. (**A**) The positive arm proteins, BMAL1, CLOCK, NPAS2, and RORs upregulate transcription of the negative arm. (**B**) Negative arm proteins, PERs, CRYs, and REV-ERBs reduce the abundance and activity of positive arm components, thereby reducing their own transcription. The reduced presence of the negative arm releases the expression of the positive arm to begin the oscillating molecular clock again. This molecular loop takes approximately 24 h to complete and is continually synchronized to tissue-relevant zeitgebers.

Due to this, light is one of the most salient time-of-day sensory signals (zeitgebers) that entrain circadian rhythms [7,8]. Photic cues are transmitted from retinal ganglia to the hypothalamic suprachiasmatic nucleus (SCN), which is regarded as the “master” circadian regulator in mammals [3,7,9]. The SCN keeps time through molecular clock genes that function in a transcriptional/translational feedback loop (TTFL) composed of a range of core proteins: brain and muscle Arnt-like protein 1 (BMAL1), circadian locomotor output cycles kaput (CLOCK), period (PER1-3), and cryptochrome (CRY1-2) with accessory proteins such as neuronal PAS domain protein 2 (NPAS2), retinoic acid-related orphan receptor alpha (RORα) and reverse ERB α and β (REV-ERBα and β) [10] (Figure 1). BMAL1, CLOCK, and NPAS2 make up the positive arm of the TTFL (Figure 1A) while PERs, CRYs, and REV-ERBs make up the negative or inhibitory arm [10] (Figure 1B). A heterodimer is formed between BMAL1 and CLOCK or NPAS2, which then activates the transcription of negative arm genes [11]. After translation, PER2 and CRY1 form a complex that returns to the nucleus to inhibit the transcriptional activity of the BMAL1 heterodimer [11]. RORα and REV-ERBs bind to the transcriptional response element for BMAL1 and NPAS2 to activate or inhibit their transcription, respectively [11]. Thus, the negative arm reduces the abundance and activity of positive arm components thereby preventing their own transcription [11]. The reduced presence of negative arm proteins then releases the expression of the positive arm to begin the TTFL again [10,11]. This molecular loop takes approximately 24 h to complete and, in the SCN, is continually synchronized to the daily light cycle [3,7,9].

Although light is a potent zeitgeber for the SCN, cells outside of the SCN are entrained to other stimuli. Indeed, the TTFL in peripheral tissues can synchronize to daily food cues [12,13,14,15,16,17,18,19]. In these tissues, clock-controlled genes (CCGs) involved in nutrient metabolism are regulated by the molecular clock in anticipation of food availability or scarcity [2]. This system maintains synchrony between the SCN and food-entrainable clocks when activity and nutrient intake align. However, the light-entrainable SCN and food-entrainable clocks can become out of sync, particularly in modern society where humans are commonly exposed to shift work and jet lag [20,21,22]. This desynchrony is especially deleterious to human health, as shift-working individuals face an increased risk of developing chronic conditions such as obesity, type-2 diabetes, and cardiovascular disease [23,24,25,26,27,28,29,30]. Therefore, the mechanisms for sensing nutrients at specific times throughout the day are a critical component of maintaining organismal homeostasis and health. In this narrative review, we will examine the current literature on time-of-day-dependent nutrient sensing mechanisms in mammalian systems and how metabolic regulatory networks keep biological time at the cellular, tissue, and organismal level.

The specific aims of this review are to firstly synthesize current evidence on time-of-day-dependent nutrient sensing mechanisms across mammalian systems, with an emphasis on molecular clock components and metabolic signaling pathways. Secondly, this review will examine how metabolic processes and nutrient-derived signals interact with the circadian clock at cellular and tissue levels to regulate gene expression, energy utilization, and physiological rhythms. Thirdly, the review will evaluate the integration of central and peripheral clocks in coordinating organismal homeostasis, including the effects of feeding/fasting rhythms and circadian desynchrony (e.g., shift work). While previous reviews have discussed circadian nutrient metabolism, this review provides conceptual support for the role of nutrients as time-of-day signaling mechanisms.

## 2. The Daily Rhythms of Metabolic Processes

One function of circadian rhythms is to initiate internal processes that anticipate daily changes in the external environment. Metabolic rhythms prepare the body to absorb nutrients when food is available and to mobilize nutrient stores during fasting [31,32,33]. This also prevents futile cycling of anabolic and catabolic processes by providing temporal separation of opposing metabolic states [33,34].

**Figure 2:**
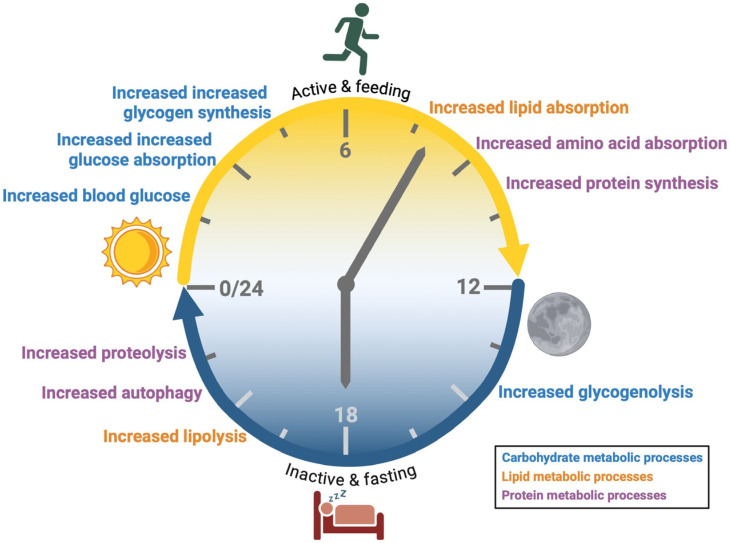
Daily Rhythms of Metabolic Processes. Metabolic rhythms prepare the body to absorb nutrients when food is available and to mobilize nutrient stores during fasting. For humans, feeding and activity occur during the light phase. The processes for glucose handing and metabolism peak in the early active phase, while lipid and protein metabolic processes peak in the late active phase. During the dark phase, humans are prepared to rest and fast. Nutrients that were stored during the light phase are therefore broken down and released to provide fuel.

In the feeding phase (light cycle for humans and dark cycle for nocturnal mammals), glucose absorption and glycogen synthesis are upregulated to accommodate the postprandial increase in blood glucose [35,36,37] (Figure 2). Similarly, lipid metabolism processes exhibit peak expression during the feeding phase; pathways related to protein absorption and synthesis are increased to process dietary fats and proteins [38,39,40,41,42] (Figure 2). Carbohydrates are more readily utilized during the early active phase while lipids and proteins are preferentially metabolized during the late active phase [35,43,44] (Figure 2). In the fasting phase (dark cycle for humans and light cycle for nocturnal mammals) glycogenolysis, lipolysis, and autophagy pathways are upregulated to mobilize stored nutrients [35,36,45] (Figure 2).

Notably, the central clock’s response to light is conserved across diurnal and nocturnal mammals [46]. Despite a reversal of the active phase, SCN neurons in both humans and nocturnal species share a photic-entrained mechanism and exhibit similar temporal expression of clock genes [47]. For example, *Per1* rhythms peak around midday for diurnal and nocturnal animals [47]. Thus, some circadian outputs are maintained independent of arousal state and remain fixed to the external day/night cycle, such as melatonin rhythms that consistently peak during the night [48,49]. With evolutionary adaptation, the transition between nocturnal and diurnal lifestyles prompted a rewiring of the relationship between the central clock and its downstream behavioral and metabolic pathways [50]. This reorganization allows the same internal timing signal to drive activity and metabolism during the day in diurnal species and during the night in nocturnal species, effectively inverting physiological outputs without altering the underlying clock mechanism [51]. 

Many of these physiological processes in the mammal occur due to anticipatory regulation of clock-controlled metabolic genes, proteins, and pathways. The discussion below will highlight these key nutrient sensory processes at the cellular, tissue, and organismal level that provide a network of metabolic timekeeping cues.

## 3. Metabolic Timekeeping in the Cell

Even before consuming a meal, the molecular machinery for metabolizing and utilizing nutrients is initiated to anticipate food intake. The molecular clock plays a key role in this response by regulating genes and processes to signal the time during which nutrients are typically available. Not only that, but nutrients themselves can regulate the speed and amplitude with which the molecular clock ticks.

### 3.1. The Clock Anticipates Nutrients

Glucose uptake is highest in the early active phase and progressively decreases to its nadir in the early inactive phase [52,53,54,55,56]. Lipid metabolism follows a similar trend, as mammals’ capacity to metabolize and store dietary lipids peaks during the active phase while lipolytic pathways increase during the inactive phase [39,41]. In the postprandial state, glucose and lipids are absorbed into circulation by the small intestine and taken up from the blood by tissues such as the liver, skeletal muscle, and adipose tissue, with each step regulated by the molecular circadian clock [52,57]. Whole body deletion of the positive circadian clock components *Bmal1* or *Clock* impairs glucose and lipid tolerance and disrupts the daily fluctuations of circulating macronutrients [58,59,60].

**Figure 3:**
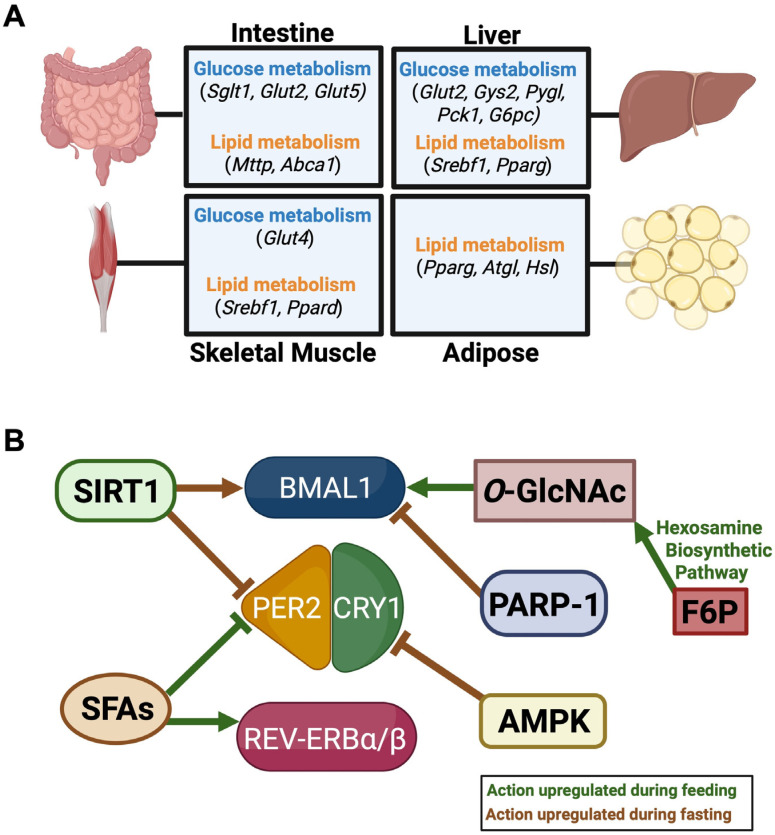
Metabolic Timekeeping in the Cell. (**A**) A non-exhaustive list of example genes and metabolic processes controlled by the molecular circadian clock in the intestine, liver, skeletal muscle, and adipose tissue. The intestinal capacity to absorb dietary carbohydrates and lipids is regulated by the molecular clock. The hepatic molecular clock facilitates the liver’s function as a macronutrient sink that can absorb, store, and release nutrients as metabolic need changes across the 24 h day. While the skeletal muscle clock plays a larger role in daily glucose homeostasis, specific lipid metabolism genes are skeletal muscle clock-controlled genes specifically in the context of exercise. In white adipose tissue (WAT), the molecular clock has a high degree of regulation over lipid metabolism and less involvement in WAT glucose handling. (**B**) A non-exhaustive schematic of proteins that signal nutrient status to the molecular clock. Cycling of substrates such as saturated fatty acids (SFAs) and fructose-6-phosphate (F6P) through adenosine triphosphate (ATP) affects the activity of enzymes and proteins like SIRT1, PARP-1, and AMPK that directly act on molecular clock components to control the speed and amplitude of circadian oscillations. Green lines and arrows indicate actions upregulated during feeding. Brown lines and arrows indicate actions upregulated during fasting.

Rhythms in whole-body macronutrient availability are maintained by tissue-specific oscillations in clock-controlled glucose metabolism genes [61,62,63] (Figure 3A). In the small intestine, the glucose transport genes *Sglt1*, *Glut5*, and *Glut2* exhibit a daily rhythm in expression [61,62,63]. Chromatin immunoprecipitation experiments identified these genes as BMAL1 targets, placing them under the control of the molecular clock [63]. Furthermore, the oscillatory expression pattern of *Sglt1* is abolished in an intestine-specific *Bmal1^−/^^−^* mouse, and knockout of *Per2* increases the concentration of carbohydrates in intestinal metabolomics analysis and circulating glucose [64,65]. Intestinal capacity to absorb dietary lipids is also controlled by the molecular clock as intestinal *Bmal1* knockouts reduce fat absorption such that animals are resistant to high-fat diet-induced obesity [66]. Conversely, mice with a mutation of intestinal *Clock* exhibit hyperlipidemia and atherosclerosis, indicating that the loss of functional CLOCK in the intestine increases lipid absorption [67,68,69] (Figure 3A).

The hepatic molecular clock facilitates the liver’s function as a macronutrient sink that can absorb, store, and release nutrients as metabolic demand varies throughout the 24 h day [70]. The hepatic glucose transporter, GLUT2, is regulated by the clock as it loses its rhythmic pattern of expression in liver-specific *Bmal1*-deficient mice [71]. To store circulating glucose, hepatic CLOCK regulates the transcriptional activity of glycogen synthase (*Gys2*) to instigate glycogenesis in the active phase [70] (Figure 3A). During the resting phase when organisms are fasting, the liver will synthesize glucose and release glycogen through gluconeogenesis and glycogenolysis, respectively [70]. These processes are regulated by hepatocyte-specific cyclic AMP-responsive element binding protein (CREBH), whose transcriptional activity is regulated by BMAL1 and RORa [72,73,74]. Molecular clock control over this transcription factor enables rhythmic expression of CREBH gluconeogenic and glycogenolytic target genes such as glycogen phosphorylase (*Pygl*), phosphoenolpyruvate carboxykinase 1 (*Pck1*), and glucose-6-phosphatase (*G6pc*) [72,73] (Figure 3A). CRY1, on the other hand, inhibits hepatic gluconeogenesis by suppressing CREBH and inhibiting FoxO1 nuclear translocation to downregulate gluconeogenic gene expression [75,76].

Multiple reports indicate that the main regulators of lipid metabolism—sterol regulatory-element binding proteins (SREBPs) and peroxisome proliferator-activated receptors (PPARs)—are under regulation of the molecular clock [77]. Rhythmicity of SREBP1 activity is controlled by BMAL1, REV-ERBs, and RORα and γ, while PER2 controls PPARγ-mediated liver lipid metabolism [74,78,79,80,81,82,83] (Figure 3A). Furthermore, metabolic state-dependent hepatic triglyceride synthesis is fine-tuned by RORα, RORγ, and REV-ERBs [74,79,82,84].

Insulin facilitates the uptake of glucose in skeletal muscle and white adipose tissue (WAT) by stimulating translocation of the insulin-sensitive glucose transporter, GLUT4, to the cell membrane [85]. In the skeletal muscle, the gene for GLUT4 (*Slc2a4*) is a CCG that exhibits decreased mRNA and protein levels upon skeletal muscle-specific *Bmal1* deletion [86,87,88] (Figure 3A). The skeletal muscle molecular clock is also an important regulator of metabolic-state-dependent glycolysis by coordinating the daily expression of key glycolytic enzymes and by utilizing the hypoxia-inducible factor (HIF1α) pathway [89,90,91]. In WAT, however, adipocyte-specific deletion of *Bmal1* does not impact *Glut4* expression or glucose handling [92]. In fact, the adipocyte-specific molecular clock appears to exert less control over WAT glucose handling than it does in the liver or skeletal muscle [93]. Although nearly a fourth of oscillating genes in WAT are glucose metabolism genes, greater physiological impacts are observed in adipocyte clock regulation of lipid metabolism genes [94]. Interestingly, lipid metabolism genes have been identified as CCGs in the skeletal muscle [88,90]. CRY1/2 in the skeletal muscle repress PPARδ-mediated lipid metabolism in response to exercise, and lipoprotein lipase (LPL) is elevated in muscle during the active phase to facilitate circulating fatty acid uptake and oxidation [36,95] (Figure 3A).

However, the skeletal muscle molecular clock appears to exert more control over carbohydrate metabolic genes than lipid metabolism genes [88,90]. This contrasts with the high degree of regulation that the molecular clock imposes on lipid metabolism in WAT. The gene expression of rate limiting and key enzymes in lipogenesis and lipolysis are directly controlled by members of the adipocyte clock [36,96,97,98,99,100]. Mutation of *Clock* and deletion of *Bmal1* decreases the expression of lipolytic regulators—adipose triglyceride lipase (ATGL) and hormone-sensitive lipase (HSL)—resulting in adipocyte hypertrophy [101] (Figure 3A). Lipogenesis is similarly controlled by the adipocyte molecular clock through diurnal regulation of PPARγ-mediated fatty acid synthesis and storage [41,102,103]. The differing clock control of carbohydrate and lipid metabolism in the skeletal muscle versus adipose tissue highlights the cell- and tissue-type specificity of CCGs.

### 3.2. Nutrients Feed the Clock

Nutrient signals and metabolic state can control clock gene expression and protein function. Indeed, time-restricted feeding studies indicate that peripheral tissue clocks can be entrained to nutrient intake and metabolic signals rather than light [16,17,32,79,104,105].

A classic marker for cellular metabolic status is the adenosine monophosphate to adenosine triphosphate (AMP/ATP) ratio [106]. In the fed, oxygen-rich state, ATP is generated from AMP and adenosine diphosphate (ADP) through means of glycolysis, the citric acid cycle or oxidative phosphorylation [106]. While in the fasted state, ATP is dephosphorylated into AMP to provide substrates for carbohydrate and lipid fuel production [106]. Substrate cycling through AMP/ATP produces several metabolic sensors that can regulate the circadian clock. Adenosine monophosphate-activated protein kinase (AMPK), for example, is activated in response to low ATP during fasting and lengthens the molecular clock period by phosphorylating and destabilizing CRY1 [107,108] (Figure 3B). With this, the period of the molecular clock appears to lengthen by signals of nutrient scarcity and shorten by signals of nutrient availability. Mammalian target of rapamycin (mTOR) is activated in the fed state, whereby increased mTOR activity shortens the circadian period in vitro and in vivo [109,110]. mTOR is negatively regulated by high AMPK activity in the fasted state, while heterozygous mTOR knockout or pharmacological inhibition has been shown to lengthen molecular clock oscillations through posttranscriptional modifications of core clock proteins [109,110].

The AMP/ATP ratio is also maintained by electron movement through coenzymes such as the redox pair, nicotinamide adenine dinucleotide (NAD+) and reduced nicotinamide adenine dinucleotide (NADH) [111]. High levels of NADH accumulate in the fed state to facilitate nutrient storage, whereas NAD+ levels rise in the fasted state to initiate oxidative pathways [111,112]. NAD+/NADH regulates the molecular clock through the ADP-ribosyltransferase (ADP-ribose) polymerase 1 (PARP-1) and the NAD+-dependent deacetylase, sirtuin 1 (SIRT1) [113,114,115,116,117]. PARP-1 regulates CLOCK:BMAL1 genome binding and response to timed food intake in peripheral tissues [108]. Loss of PARP-1 increases CLOCK:BMAL1 DNA binding and impairs the liver clock’s ability to entrain to food intake [117] (Figure 3B). Elevated NAD+ in fasting increases SIRT1 deacetylation of BMAL1 while inhibiting the histone acetyltransferase function of CLOCK [113] (Figure 3B). NAD+-dependent SIRT1 activity also deacetylates PER2 thereby initiating PER2 degradation and enhancing BMAL1 chromatin binding [114,115] (Figure 3B). The SIRT1-mediated effects of NAD+ on molecular clock factors enhance the amplitude and speed of CCG expression, which has been found to restore dampened circadian rhythms in aged animals and have a positive effect on metabolism [115,118,119,120]. However, the response to NAD+ levels is dependent on time of day, as NAD supplementation prior to the active phase ameliorates the metabolic effects of a high-fat diet (HFD) while NAD administration before the rest phase increases HFD-induced insulin and glucose insensitivity, body weight, and hepatic inflammation [112].

Macronutrients themselves may act as nutrient signals that influence the circadian clock. During fed-state glycolysis, fructose-6-phosphate (F6P) may enter the hexosamine biosynthetic pathway (HBP) via glutamine-fructose-6-phosphate transaminase 1 (GFPT1) [121]. The byproduct of F6P through the HBP is *O*-linked b-D-N-acetylglucosamine (*O-*GlcNAc) [121]. BMAL1 and CLOCK are *O*-GlcNAcylated in response to meal timing [122]. Furthermore, the key regulatory enzyme in the *O*-GlcNAc production pathway promotes expression of CCGs and regulates oscillation of clock genes by regulating ubiquitination of BMAL1 and CLOCK [123] (Figure 3B). Non-obesogenic doses of the saturated fatty acids (SFAs) palmitate and oleate interfere with BMAL1 transcriptional activity in hepatocytes and adipocytes [124,125]. In hepatocytes, palmitate inhibits PER2 nuclear translocation and increases REV-ERBα repression of *Bmal1* [124] (Figure 3B).

## 4. Metabolic Timekeeping in the Tissue

Circadian regulation of metabolism emerges not only from cell-autonomous molecular clocks and cellular nutrient sensation, but from dynamic communication within and between tissues and organs [126]. Intercellular coupling within the SCN and coordinated signaling between central and peripheral clocks ensure the temporal alignment of metabolic processes with environmental and behavioral rhythms [117]. Disruption at any level of this network—cellular, tissue-specific, or systemic—can propagate broadly to impair metabolic homeostasis.

### 4.1. Cell–Cell Communication

A classic example of circadian cell–cell communication is observed in cells of the SCN. Intercellular coupling across the SCN oscillator network can compensate for single gene deficiencies and sustain circadian rhythmicity in the tissue [127]. Individual SCN neurons do not oscillate in unison with one another by default and are arranged topographically according to their differential phases [128]. Rather, disparate SCN neurons establish alignment in the timing of their Na^+^-dependent action potentials, with interneuronal synchrony facilitated by peptide signaling to produce and maintain rhythmic expression [128,129,130].

Outside of the neuron, SCN astrocytes also exhibit 24 h rhythms that regulate oscillations in SCN neurons [131]. Manipulation of the astrocytic TTFL has confirmed the necessity of astrocytes for SCN timekeeping and maintenance of behavioral rhythms [121,122,123]. Namely, global deletion of *Bmal1* from astrocytes results in longer circadian periods of rest or activity in mice [132,133]. When considering the role of hypothalamic astrocytes in metabolic timekeeping, it is possible that their many nutrient-sensing mechanisms provide a crucial link for coordinating central and peripheral oscillators [134]. Specifically, astrocytes are sensitive to glucose and fatty acids and express receptors for insulin, insulin-like growth factor-1 (IGF-1), leptin, thyroid hormone, and glucocorticoids [134]. As a result, astrocytes and their local neurons are heavily attuned to rhythms of feeding and fasting for the maintenance of brain metabolic homeostasis [134]. During periods of fasting, hypothalamic astrocytes facilitate the uptake of ketone bodies into the cell, as neurons switch from using glucose to ketones for their primary energy source [134]. To support that this metabolic activity is coupled to the astrocyte circadian clock, astrocyte-specific deletion of *Bmal1* is sufficient to impair glucose homeostasis [135].

It was originally believed that cell–cell timekeeping was solely conducted by the SCN in response to environmental light/dark cycles [7]. In recent decades, it has become evident that cell type-specific clocks also communicate in peripheral organs, such as the liver [79]. Notably, a hepatocyte-specific deletion of REV-ERBα and β impairs gene expression and circadian rhythmicity in hepatic endothelial cells and Kupffer cells [79]. The liver’s daily transcriptome is significantly reprogrammed after REV-ERβ deletion, which in turn alters rhythms of lipid synthesis and increases triglyceride levels [79]. Hepatocyte-specific knockout of Bmal1 produces similar results but also affects perivascular adipocyte function by altering metabolic gene expression and subsequently decreasing blood pressure [136]. In comparison, deletion of *Bmal1* in pancreatic β-cells generates downstream effects on daily glucose homeostasis and insulin secretion, leading mice to develop diabetes [137]. This suggests that the loss of the cell type-specific clocks poses wide-ranging effects on intercellular signaling and metabolic physiology.

### 4.2. Organ Crosstalk

**Figure 4:**
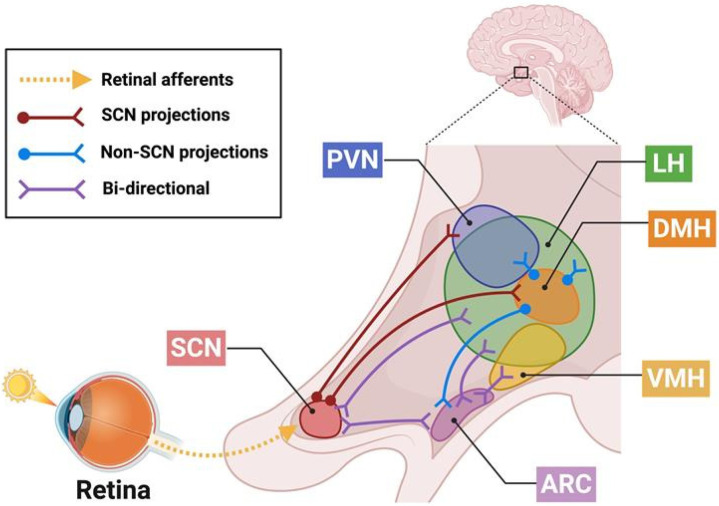
Central Clocks. Schematic of interaction between the central clocks of the hypothalamus after the suprachiasmatic nucleus (SCN) receives photic inputs from retinal ganglia. The SCN projects directly to the paraventricular nucleus (PVN) and dorsomedial hypothalamic nucleus (DMH) and communicates bidirectionally with the lateral hypothalamus (LH) and arcuate nucleus (ARC). The DMH acts as an intermediate nucleus for light-entrained signaling from the SCN, relaying information to the PVN, LH, and ARC. In addition to its reciprocal connections with the SCN, the ARC bidirectionally communicates clock-controlled information with the LH and ventromedial hypothalamus (VMH).

After the SCN receives photic inputs from the retina, information is firstly transmitted to other hypothalamic nuclei and integrated into hormonal and autonomic signals to be received by peripheral organs [126]. Non-SCN clocks of the brain’s dorsomedial hypothalamic nucleus (DMH), ventromedial hypothalamus (VMH), paraventricular nucleus (PVN), and arcuate nucleus (ARC) coordinate with the SCN to adjust and maintain energy homeostasis for the regulation of feeding behaviors [138] (Figure 4). Like peripheral clocks, these hypothalamic nuclei can be synchronized by external rhythms coupled to feeding/fasting schedules [138]. Despite this, roughly 8–10% of transcriptomes throughout the body’s tissues are controlled by the SCN, highlighting the importance of the light-entrained circadian clock for global functioning and its sustained central-peripheral action [139,140]. In support of this, bilateral SCN lesions lead to total arrhythmia in circadian food intake [141,142]. Thus, optimal conditions for metabolic homeostasis occur when there is synchrony between the SCN and peripheral clocks.

#### 4.2.1. Intestine

Under the SCN clock, tissue-specific peripheral clocks can be organized hierarchically, ranked in order of their entrainment to feeding and fasting cues [143]. Recent evidence has demonstrated the high-ranking role of the intestinal clock as an inter-organ metabolic timekeeper [144]. Although the cell-autonomous liver clock was originally thought to dominate many downstream metabolic rhythms, hepatic timing for glucose production and lipid synthesis are largely determined by time-encoded signaling from the intestine [144]. The intestine utilizes nutrients and metabolites, such as polyunsaturated fatty acids (PUFAs), to transmit feeding-entrained rhythms to the liver [144]. When the intestinal clock is impaired (via *Bmal1* or REV-ERBα deletion), the liver’s rhythmic transcriptome is widely reprogrammed, causing phase shifts for lipogenesis and glucogenesis regardless of meal timing [144]. This demonstrates that feeding/fasting cycles and nutritional cues alone are not sufficient for setting the liver’s daily programs, but rather that the intestine is a necessary intermediary for food-based entrainment across organs.

#### 4.2.2. Liver

**Figure 5:**
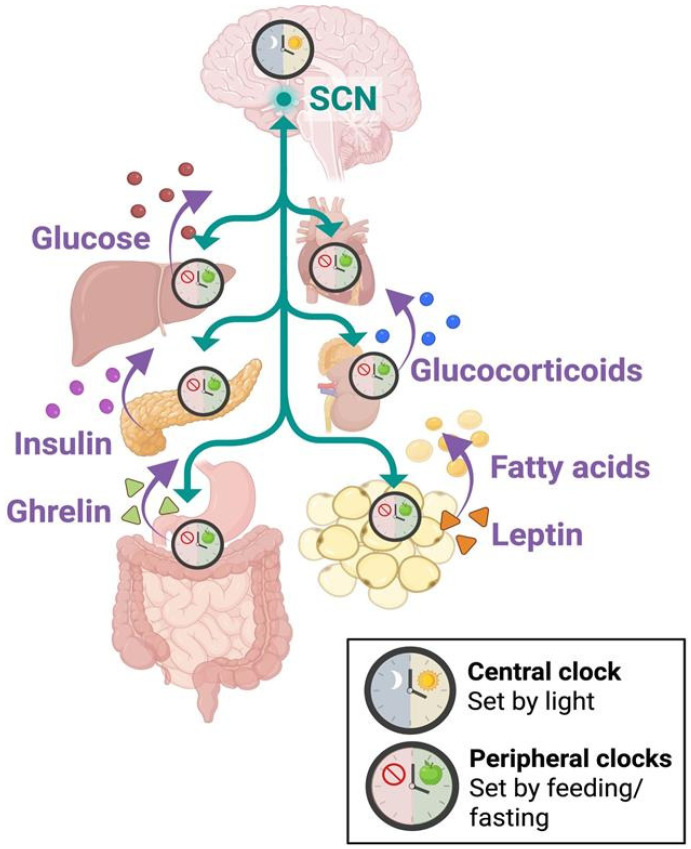
Peripheral Tissue Clocks. From the hypothalamus, the central SCN clock expresses circadian rhythms entrained by the daily light/dark cycle. SCN light-entrained signals project downstream to set the clocks of peripheral tissues and metabolic organs, such as the heart, liver, adrenal glands, pancreas, gut, and adipose tissue. External cues of feeding and fasting modulate the rhythmic expression of metabolic activity in peripheral tissues, e.g., glucose release from the liver, glucocorticoids from the adrenal glands, insulin from the pancreas, fatty acids and leptin from adipose tissue, and ghrelin from the stomach. Peripheral metabolic cues are communicated back to the SCN to optimize circadian alignment between central and peripheral clocks. Purple arrows indicate hormonal signals. Teal arrows indicate neuronal signals.

Alongside normal functioning of the intestinal clock, the liver employs signals from feeding and fasting to drive rhythms of the hepatic transcriptome and thereby influence ensuing metabolic activity [44] (Figure 5). For example, human blood glucose concentrations—regulated in part by the liver—typically remain stable during periods of fasting [145]. When coordinating with central clock oscillations, there is a direct relationship between the SCN and hepatic outputs associated with insulin sensitivity and glucose production [145]. To reinforce this, ablation of the SCN results in elevated blood glucose as well as increased adipose tissue [142]. Glycemia and fat mass are then proportionately increased when hypothalamic lesions are extended to include the adjacent VMH and PVN [142]. When rhythmic output of blood glucose is altered—as seen with hepatocyte-specific deletion of *Bmal1*—this is sufficient to also affect phase entrainment of adipose and lung tissues [146]. As disruptions to the hepatic clock strongly modulate the rhythmicity of distant peripheral tissues, this emphasizes the liver’s high-order role in preserving metabolic timekeeping between organs.

#### 4.2.3. Pancreas

The pancreas acts as both an endocrine and exocrine gland by releasing hormones and enzymes that regulate daily rhythms of digestion and glycemia [147] (Figure 5). When the pancreatic clock is disrupted by *Bmal1* deletion, glucose tolerance and insulin secretion become severely defective, even when feeding patterns and insulin content are normal [148]. Conversely, normal circadian rhythms of the endocrine pancreatic clock can be affected by reversed restricted feeding, with rhythmic clock gene expression shifting to correspond with changes in feeding times [149]. As meal timing entrains the rhythm of insulin secretion, this interacts with the peripheral clock of the liver. Hepatic activity then becomes synchronized to the endocrine pancreatic clock for management of glucose homeostasis [150]. Despite this, the exocrine pancreas clock does not shift with mistimed feeding schedules and can become arrhythmic with the uncoupling of insulin and corticosterone (adrenal gland) rhythms [149]. Furthermore, misaligned feeding can uncouple these peripheral clocks of the pancreas and liver from the central SCN clock [151]. Specifically, the pancreas communicates daily insulin and glucagon levels to the hypothalamus for the circadian regulation of appetite and energy metabolism [152]. To complete the bidirectional loop, melatonin and melanocortin signals between the brain and periphery to influence pancreatic clock gene expression and timing for hormone secretion [153].

#### 4.2.4. Adipose Tissue

The adipocyte clock is another essential driver for coordinating metabolic rhythms and energy homeostasis between central and peripheral tissues [87,90,91] (Figure 5). Adipocyte-specific *Bmal1* deletion leads to systemic shifts in feeding rhythms, with mice becoming obese due to increased food intake during their inactive phase (light) and lower food intake during their active phase (dark) [92]. Disruptions in adipocyte timekeeping propagate to behavior, as feedback from adipose tissue is cyclically communicated to the brain’s hypothalamus [91]. Lipid metabolites, particularly PUFAs, circulate in peripheral adipose tissue to act as metabolic signals for the hypothalamus [91,154] (Figure 5). Based on these cues, central clocks in the brain adjust the timing of feeding behaviors, thereby entraining metabolic processes across multiple organs and coordinating whole-body metabolic rhythms.

## 5. Metabolic Timekeeping in the Body

### 5.1. Hormonal

Through tightly regulated daily rhythms and acute responses to feeding or fasting, hormonal signals synchronize central and peripheral oscillators to ensure that metabolic processes occur at optimal times. It is well established that hormones such as insulin, insulin-like growth factor-1 (IGF-1), glucocorticoids, leptin, and ghrelin follow 24 h rhythms and exert varying degrees of control over the body’s circadian clocks [143,145] (Figure 5).

#### 5.1.1. Insulin, IGF-1, and Glucocorticoids

With their receptors ubiquitously expressed throughout most tissues of the body, insulin and related IGF-1 have been identified as robust temporal cues for communicating feeding patterns to circadian clocks across diverse cell types [155]. To regulate blood glucose changes that occur with feeding, circulating insulin and free IGF-1 increase acutely following carbohydrate and protein/fat intake, respectively [156]. These hormonal signals induce rapid synthesis of PER proteins via intracellular mTOR activation, with insulin being sufficient to shift the phase, period, and amplitude of oscillating rhythms [155]. Although the central SCN clock is more resistant to food-associated entrainment, peripheral clocks appear to synchronize their daily gene expression and rhythmic outputs to align with the reception of postprandial insulin and IGF-1 [157,158,159]. These findings position insulin and IGF-1 as systemic timekeepers that translate time-of-feeding into phase alignment of peripheral clocks, effectively coordinating daily metabolic physiology with nutrient availability.

Glucocorticoid hormones—such as cortisol in humans and corticosterone in rodents—are released from the adrenal gland in adherence to a strong circadian rhythm (Figure 5), as levels are lowest during the inactive phase and rise to a peak just before the active phase [160]. With this, daily glucocorticoid secretion is driven by the SCN to align with the 24 h light/dark cycle. However, glucocorticoid binding alone has the capacity to directly reset the timing of clock gene expression (e.g., *Per*) in major metabolic organs, such as the liver, kidney, and heart [161]. As a result, glucocorticoids induce phase-shifting in peripheral clocks without affecting the SCN, demonstrating their potency to synchronize body clocks and structure metabolic activity across the day. Glucocorticoid rhythms enable whole-body mobilization of energy stores (e.g., glucose, fats, proteins) to be optimally timed for when the organism is most active [162,163].

When examining the relationship between glucocorticoids and insulin, their signals interact antagonistically, as both compete for hierarchical control over the peripheral circadian clock. While insulin works to align peripheral clocks with time-of-feeding, glucocorticoids attempt to counteract this effect by maintaining synchrony with the light-entrained SCN. Despite insulin’s ability to rapidly induce *Per* after feeding, glucocorticoid receptor signaling has the potential to suppress *Per* transcription and bias clock gene expression toward SCN rhythms [164]. This mechanism inhibits food-entrained phase-shifting and prevents excessive uncoupling of peripheral clocks from the central SCN oscillator [164]. Importantly, glucocorticoid hormones help reduce whole-body desynchrony following mistimed eating by reinforcing metabolic harmony across all tissues.

#### 5.1.2. Leptin and Ghrelin

Leptin is an adipokine that acts as a satiety signal to communicate sufficient energy stores from adipose tissue to the hypothalamus [155] (Figure 5). Leptin signaling peaks postprandially to suppress appetite and promote energy expenditure [165]. In contrast to insulin or glucocorticoids, leptin can modulate circadian rhythmicity in the SCN clock [166]. Administering leptin treatment directly to SCN tissue that is isolated in vitro phase-advances the clock in a dose-dependent manner, i.e., shifting SCN oscillations to occur ~1–3 h earlier [166]. To maintain energy homeostasis, leptin interacts with neuronal clocks to increase or decrease feeding behavior at appropriate times of day [167]. Mice deficient in leptin (*ob/ob*) exhibit increased hedonic feeding during their inactive phase, suggesting that leptin signaling helps coordinate time-of-feeding preference with the organism’s active phase [167]. *Per1/2* or *Bmal1* knockout mice show similar arrhythmia in homeostatic (energy-driven) and hedonic (pleasure-driven) feeding, which indicates the necessity of clock genes for leptin to appropriately time feeding behavior based on daily metabolic needs [167]. Interestingly, *Bmal1* deletion in leptin receptor-expressing neurons causes feeding behavior to be less driven by palatable foods, meaning that the central clocks of leptin-sensing reward circuits time their rhythms with metabolic signals to promote reward-based feeding [167].

In opposition to leptin, the orexigenic hormone ghrelin is released preprandially from the stomach to stimulate appetite and increase feeding behavior [168] (Figure 5). Both ghrelin and leptin signal from peripheral tissues to act inversely on hypothalamic neurons for the cyclical regulation of hunger and fullness [158]. When SCN slices are treated with ghrelin in vitro, the SCN clock behaves similarly to leptin treatment, with rhythms phase-advanced by approximately 3 h following a shift in *Per2* gene expression [169]. However, this response is not conserved in vivo when mice are injected with ghrelin on a normal feeding schedule, suggesting that the light-entrained rhythms of the SCN override ghrelin under normal conditions [169]. After 30 h of food deprivation, mice injected with a ghrelin analog (growth hormone-releasing protein-6) exhibit phase-advanced activity [169]. With this, the body’s circadian clocks appear to become more susceptible to ghrelin’s “hunger” signals in periods of fasting, and ghrelin may function as a secondary timekeeper when normal cues of food availability are disrupted. Nonetheless, it is important to note the direct functional link between SCN neurons expressing ghrelin receptors (GHSR), circadian regulation of hunger, and time-dependent feeding behavior [170]. In other words, ghrelin provides a mechanistic bridge through which the master SCN clock actively gates feeding behavior according to the time of day [170].

Several studies have identified ghrelin-secreting cells as potential food-entrainable oscillators (FEOs) that regulate the body’s circadian systems [69,171,172,173]. In particular, oxyntic cells of the stomach exhibit rhythmic co-expression of Per1, Per2, and ghrelin in 24 h light–dark cycles as well as in constant darkness with ad libitum feeding, which points to their persistence without external light cues [173]. Oxyntic clock gene expression and ghrelin release also retain rhythmicity in conditions of prolonged fasting (48 h), but the circadian clock phase becomes dependent on time of food availability [173]. Similar results are seen in intestinal L-cells for the circadian secretion of the incretin hormone glucagon-like peptide-1 (GLP-1), where release is correlated with metabolic cues (insulin) and depends on time-of-feeding [174]. Together, these rhythmic secretion profiles exemplify how gut peptide-expressing cells work to synchronize metabolic processes, nutrient availability, and the circadian clock.

When the clock is disrupted by *Bmal1* deletion, normal daily rhythms are abolished in mRNA expression of ghrelin and ghrelin-activating enzymes, plasma ghrelin levels, and food intake [175]. These *Bmal1*-deficient mice are also unable to adapt to restricted feeding schedules, which yields fatal results following severely reduced food intake and weight loss [176]. These findings underscore that intact circadian clock signaling is essential for ghrelin-mediated feeding and the maintenance of energy balance. Furthermore, rhythmic ghrelin release conveys information about nutritional state to central and peripheral clocks, using food availability as an entraining cue to align behavioral, metabolic, and circadian physiology with the appropriate time of day.

### 5.2. Neural

Although the majority of outgoing SCN transmissions are governed by external light cycles, the brain’s master clock is host to multiple subpopulations of neuronal oscillators that receive an array of nonphotic input from neighboring hypothalamic nuclei [177] (Figure 4). Among these afferents is circadian information pertaining to the body’s various metabolic and physiological processes, including hormone levels, nutritional availability, and feeding/fasting behaviors [178,179,180,181]. Reciprocal connections from the central SCN to adjacent hormone-sensitive regions allow the brain’s neural circuits to engage in complex multidirectional communication with peripheral systems and collaboratively regulate daily rhythms of metabolic activity [1].

#### 5.2.1. Arcuate Nucleus

**Figure 6:**
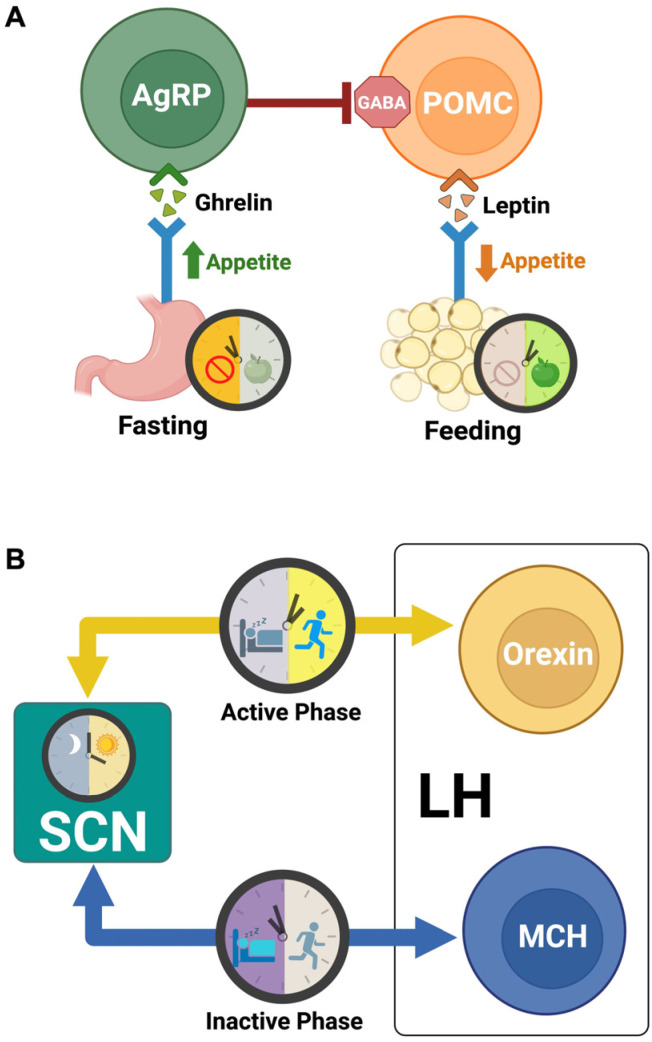
Neural Dynamics for Metabolic Timekeeping. (**A**) Hypothalamic neurons that make up the homeostatic feeding circuit in the arcuate nucleus. Agouti-related peptide (AgRP) neurons receive ghrelin from the stomach during periods of fasting. Pro-opiomelanocortin (POMC) neurons receive leptin from adipose tissue during feeding and functionally suppress appetite. AgRP neuron outputs, including GABA, work to stimulate appetite and promote feeding behavior by directly inhibiting POMC neurons. (**B**) Neuronal interactions between the suprachiasmatic nucleus (SCN) and lateral hypothalamus (LH). LH orexin neuron activity peaks during the active phase (daytime for humans, nighttime for rodents). Melanin-concentrating hormone (MCH) neuron activity peaks during the inactive/resting phase. Both neurons communicate bidirectionally with the SCN to manage energy homeostasis and feeding/fasting rhythms in alignment with daily light/dark cycles.

The hypothalamic ARC is composed of two main neuron subtypes that complete the core homeostatic feeding circuit: agouti-related peptide (AgRP) neurons that co-express neuropeptide Y (NPY) and pro-opiomelanocortin (POMC) neurons that co-express cocaine- and amphetamine-regulated transcript (CART) [181]. In tandem with the antagonistic actions of ghrelin and leptin, AgRP neurons are activated during fasting to stimulate appetite, and POMC neurons become active with feeding to suppress appetite [171] (Figure 6A). Each neuron population works to functionally oppose the other. POMC neurons release α-melanocyte-stimulating hormone (α-MSH) and activate melanocortin 4 receptors (MC4R) after a meal, whereas AgRP neurons block α-MSH binding to MC4R and directly inhibit POMC neurons through the release of orexigenic NPY and GABA [182] (Figure 6A).

Rather than track energy deficits as they occur, AgRP neurons integrate past time-of-feeding information with current metabolic cues to effectively predict daily patterns in meal timing [183]. Based on this, AgRP neuron activity peaks around expected feeding times and will decline outside the predictive window, regardless of the animal’s hunger or nutrient availability [183]. Although AgRP neuron rhythms are primarily attuned to light–dark transitions in ad libitum feeding conditions, their oscillations continue even in constant darkness, indicating that light is not a prerequisite for their sustained rhythmic activity. Instead, AgRP neurons adapt their timing to align with novel or restricted feeding schedules [183]. This is achieved through a rhythmic feedforward circuit, wherein ARC AgRP neurons are acutely excited by thyrotropin-releasing hormone (TRH) neurons of the DMH, which are firstly activated by SCN projections [184]. Ablation of the SCN then abolishes circadian rhythms in AgRP neurons, as this disrupts intermediate signaling from the DMH [184].

As seen in the AgRP neuronal clock of nocturnal rodents, canonical clock genes are differentially expressed between morning (inactive phase) and night (active phase), where *Bmal1* is increased and *Rev-erbβ, Per1*, and *Per2* are decreased in the morning [185]. Immediate early genes (e.g., *Fosl2*) as well as genes encoding DNA-and RNA-binding proteins are also decreased in the morning, indicating that the AgRP clock compels neuronal activity more directly in the evening, and thereby reinforces feeding during the nocturnal animal’s active phase [185]. When *Bmal1* is knocked out specifically in AgRP neurons, these transcriptional rhythms are significantly diminished, while leptin-responsive genes are shifted and overexpressed [185]. Nutrient-sensitive mTORC1 is also needed to maintain daily rhythms of AgRP and NPY expression, as evidenced in mTORC1-deficient mice [186]. Interestingly, even when mTORC1 signaling is manipulated in the ARC and AgRP rhythms are lost, feeding behaviors remain intact [186].

When examining the preservation of feeding rhythms, it is important to consider the influence of other neuron types that regulate circadian outcomes. In contrast to AgRP neurons, disruption of mTORC1 signaling in POMC neurons translates to behavioral changes in food intake [187]. When POMC expression is specifically knocked out from hypothalamic neurons, feeding patterns are altered in the form of larger meals and longer inter-meal intervals [188]. Additionally, disruption of the liver clock via REV-ERBα/β deletion induces downregulation of POMC and CART at the end of the animal’s active phase, suggesting the necessity of rhythmic hepatic outputs for maintaining the ARC transcriptome and priming anorectic signaling before the inactive phase [189]. However, at the core of POMC neuronal activity, their diurnal rhythms are chiefly determined by external light cycles and require intact signaling from the SCN [190]. Like AgRP neurons, POMC neurons display greatest immediate early gene expression during the animal’s active phase, aligned closely with light onset and offset [191,192]. When measuring POMC neuronal outputs, α-MSH is also highly rhythmic with a diurnal peak, but this is blunted when clock protein *Per2* expression is impaired and presents with arrhythmic feeding throughout both light and dark phases [193].

In correspondence with light-entrained activity of the SCN, histamines are received by both POMC and AgRP neurons to bias the hypothalamic feeding circuit toward satiety during the animal’s active phase [194,195]. Histaminergic neurons of the tuberomammillary nucleus (TMN) indirectly receive circadian input from the SCN and subsequently modulate the animal’s arousal state [196]. In turn, SCN neurons also express histaminergic receptors that functionally inhibit SCN activity [196]. While histamine H1 receptor activation correlates with suppressed food intake and reduced body weight, H3 receptors expressed throughout the ARC (as well as the VMH, DMH, and PVN) mediate orexigenic rhythms and are valued as a potential therapeutic target for anti-obesity drugs [197,198,199].

Taken together, there is growing evidence to support the importance of ARC neurons and their related circuits in coordinating metabolic synchrony between feeding behaviors and daily light cycles. Despite the functional link between the SCN and the meal-predictive rhythms of ARC neurons, ablation of SCN light-controlled activity does not eliminate food anticipatory behavior in the mammal [200]. Especially seen in rodents, the ~1–3 h before a scheduled feeding show a marked increase in arousal, locomotion, hormone release, and neural activity that are independent of the SCN clock [201]. Rather, it is more likely that food anticipatory activity (FAA) emerges from a distribution of FEOs that respond to a myriad of metabolic rhythms, including appetite-regulating signals of ghrelin and leptin, circadian hormones (insulin, IGF-1, glucocorticoids), and prandial cues (glucose, fatty acids) [202]. While some of these metabolic cues act through AgRP and POMC neurons of the ARC, FAA is borne from the reciprocal and time-dependent interactions of hypothalamic nuclei, motivational/reward circuits, and arousal systems (histamine and orexin) that optimize metabolism before eating [203].

#### 5.2.2. Lateral Hypothalamus

By receiving projections from both the SCN and ARC, the lateral hypothalamus (LH) is another key integrator of the body’s circadian rhythms and metabolic activity [138] (Figure 6B). Specialized neurons of the LH include those expressing orexin (also called hypocretin) and melanin-concentrating hormone (MCH) neurons [183]. Functionally, both orexin and MCH neurons are attributed to systemic regulation of daily sleep–wake cycles as well as feeding patterns and energy homeostasis [204]. Within the last century, the LH itself has gained a reputation for being the brain’s “feeding center,” with numerous studies documenting the immediate feeding response elicited by direct LH stimulation [205,206,207,208,209].

In nocturnal animals such as rodents, orexin neuron activity peaks during the active (dark) phase and falls during inactivity [210] (Figure 6B). When animals are exposed to short bursts (6 h) of darkness during the subjective day, this is sufficient to activate orexin neurons and phase-advance behavioral rhythms [210]. Expression of orexin precursor, prepro-orexin, and orexin receptors also follow strong 24 h rhythms that highly correlate with clock gene (i.e., *Bmal1*) expression [211]. Prepro-orexin mRNA levels are elevated during periods of fasting, coinciding with greater quantities of excitatory synapses on orexin neurons [212,213]. Antagonism of orexin receptors inhibits overall food intake and reduces hedonic feeding behavior [214,215]. Due to their important role in modulating arousal and wakefulness, orexin neurons are believed to optimize the timing of food searching and reward-based feeding when the animal is most active [216,217]. In the same vein, orexin neurons modulate rhythmic glucose activity in a time-dependent manner that facilitates whole-body homeostasis [218].

While orexin neuron rhythms are predominantly guided by incoming SCN signals, it is also understood that orexin outputs shape the activity of the SCN [219] (Figure 6B). For one, the SCN expresses orexin receptors that are upregulated during the animal’s active phase [220]. When administered in vitro, orexin suppresses *Per1*-expressing SCN cellular clocks [220]. Paired with NPY, orexin exerts an additive effect on hyperpolarizing SCN neurons and phase-shifting clock expression of *Per1* [220]. Through bidirectional control of central circadian activity, orexin neurons serve as a neural interface between the body’s oscillatory rhythms and metabolic/behavioral processes.

Analogous to the complementary actions of AgRP and POMC neurons in the ARC, orexin neuron activity is balanced by MCH neurons that are intermingled in the LH. Organisms deficient in orexin neurons frequently develop obesity, whereas ablation of MCH neurons is associated with reduced body weight and fat mass [221]. Under normal circumstances, MCH neurons work to promote energy conservation, with neuronal activity peaking during the animal’s inactive phase, while orexin neurons discharge during waking states [222] (Figure 6B). This rhythmic interplay between the two neuron populations allows for circadian management of energy homeostasis. Despite their differences, activation of either orexin or MCH neurons in the LH has been shown to increase food intake and feeding behavior [223,224,225]. The mechanism by which this occurs is timing-dependent and is influenced by dopaminergic reward systems [226]. Specifically, MCH neurons become active during the time of feeding and thus have the capacity to prolong meal duration and feeding rhythms [226,227]. To support MCH activity in alignment with the animal’s inactive versus active phase, the SCN transmits direct glutamatergic signals to MCH neurons for appropriate regulation of arousal throughout the light/dark cycle [228]. As a result, MCH outputs exhibit diurnal variation, with peptide signaling significantly higher at the end of the light phase (inactive) compared to the end of the dark phase (active) [229]. MCH neurons also project back to the SCN, evidenced by the expression of MCH receptors in SCN neurons [178] (Figure 6B). When either MCH or orexin are applied to cultured SCN slices, the circadian period is shortened, causing the clock to run faster [178]. Conversely, removing these LH neurons results in a longer circadian period, indicating the need for endogenous MCH and orexin to sustain 24 h rhythmicity [178]. In addition, MCH/orexin signaling alters intracellular Ca^2+^ and cAMP in SCN neurons, with effects that depend on the time of day [178] (Figure 6B).

The lateral hypothalamus is a dynamic, bidirectional hub through which circadian timing and metabolic state are continuously integrated [167,183]. By coupling SCN temporal cues with ARC-mediated energy signals and relaying this information through the opposing yet complementary actions of orexin and MCH neurons, the LH ensures that arousal, feeding, and energy balance are appropriately aligned with the external light/dark cycle. Disruption of this network therefore has the potential to uncouple circadian rhythms from metabolic regulation, emphasizing the hypothalamus’ central role in maintaining homeostasis across behavioral and physiological domains [167,183].

## 6. Conclusions and Future Directions

The circadian clock and metabolism are inextricably linked. The molecular clock regulates the expression of key metabolic genes and processes to prepare the body for feeding and fasting [2]. On the other hand, metabolic signals indicating nutrient availability or scarcity can directly regulate the expression and function of circadian clock components [16,17,32,79,104,105]. This relationship may be observed at the cellular, tissue, and organismal level and is essential for maintaining homeostasis and health.

The implications arising from the works discussed in this narrative review are particularly relevant for individuals that are required to work in environments that are at odds with their endogenous circadian rhythms. Shift-working individuals, who are often eating at the “wrong” time of day, are inordinately affected by chronic diseases such as obesity, cardiovascular disease, and other metabolic disease [27,28,30,105]. This is in part due to desynchrony between daylight and food intake as nutrient and metabolic signals that conflict with light-entrained circadian rhythms can exacerbate certain disease states [20,21,22].

As nearly 25% of adults work during non-traditional hours, more research is needed to develop targeted interventions and evidence-based strategies that mitigate circadian misalignment by harnessing the unique biology of nutrient sensing pathways and metabolic regulatory networks that interact with the circadian clock [230]. Together, this body of work highlights how circadian timekeeping is achieved at the cellular, tissue, and organismal level through nutritional and metabolic sensory networks. Specifically, this review draws from current literature to provide a novel perspective for nutrients as time-of-day signaling mechanisms, acting both in synchrony with and independent from light-controlled processes.

## 7. Methods

To compose this narrative review, the authors synthesized literature on nutrients as timekeeping signals at the molecular, cellular, tissue, and organism level. The literature selection strategy—including databases, keywords, and publication date range—is outlined in Table 1. Additional articles found outside of the specified search criteria were manually selected from the relevant references/citations presented in the qualifying literature (also known as “snowballing” or “citation chaining”) to trace the development of circadian concepts over time or to bridge gaps in the evidence. Articles selected from the provided search results were then chosen based on relevance of the title and abstract with respect to this review’s aims.

### Limitations

This narrative review is subject to several limitations. As a non-systematic review, literature selection may reflect author bias, the search was not exhaustive, and relevant studies may have been omitted. Most works cited present evidence from preclinical models, chiefly using nocturnal rodent models. This limits direct translation to human physiology, particularly given differences in diurnal biology and experimental conditions. Additionally, differences in the methodology across cited studies (e.g., genetic models, omics analyses, timing of sample collection) further constrain direct comparisons and may contribute to inconsistencies in interpretation. Circadian studies are limited by temporal sampling, and thus may not fully represent total 24 h dynamics of a biological rhythm. With this, the conclusions drawn from this review should be viewed as a synthesis of current knowledge rather than a comprehensive and definitive framework.

## Figures and Tables

**Table 1:** Literature Search Strategy. Literature searches were performed using exact search strings in both Google Scholar and PubMed databases. * indicates a wildcard Boolean operator used to represent multiple characters or work endings to widen our literature search.

Databases Searched	Publication Date Range	Keyword Search String
Google Scholar; PubMed	2010–2026	(circadian OR rhythm) AND (“suprachiasmatic nucleus”) AND (TTFL OR oscillator) AND (Bmal1 OR Cry * OR Per * OR Rev-ERB * OR ROR* OR NPAS2)
Google Scholar; PubMed	2010–2026	(circadian OR clock) AND (feeding OR “food intake” OR fasting) AND (metaboli *) AND (peripheral OR liver OR pancreas OR adipose OR gut OR intestines OR “food-entrainable oscillator”)
Google Scholar; PubMed	2010–2026	(circadian OR clock) AND (feeding OR “food intake” OR fasting) AND (metaboli *) AND (glucose OR glycogen OR carbohydrates OR lipids OR “fatty acids”)
Google Scholar; PubMed	2010–2026	(circadian OR clock) AND (feeding OR “food intake” OR fasting) AND (metaboli *) AND (IGF * OR glucorticoid OR leptin OR ghrelin)
Google Scholar; PubMed	2010–2026	(circadian OR clock) AND (feeding OR “food intake” OR fasting) AND (metabolic) AND (“lateral hypothalamus” OR “arcuate nucleus”) AND (AgRP OR POMC OR orexin OR MCH)
Google Scholar; PubMed	2010–2026	(circadian OR clock) AND (“restricted feeding” OR “reverse feeding” OR “food intake” OR fasting) AND (metaboli *) AND (“shift work”) AND (disease OR obesity OR diabetes)

## Data Availability

No new data were created of analyzed in this study. Data sharing is not applicable to this article.

## Abbreviations

The following abbreviations are used in this manuscript:

SCN	Suprachiasmatic nucleus
TTFL	Transcription/translation feedback loop
BMAL1	Brain and muscle Arnt-like protein 1
CLOCK	Circadian locomotor output cycles kaput
PER	Period
CRY	Cryptochrome
NPAS2	Neuronal PAS domain protein 2
ROR	Retinoic acid-related orphan receptor
REV-ERB	Reverse of ERB
CCG	Clock-controlled gene
GLUT2	Glucose transporter 2
*Gys2*	Glycogen synthase
CREBH	Cyclic AMP-responsive element binding protein
*Pygl*	Glucogen phosphorylase
*Pck1*	Phosphoenolpyruvate carboxykinase 1
*G6pc*	Glucose-6 phosphatase
SREBP	Sterol regulatory-element binding protein
PPAR	Peroxisome proliferator-activated receptors
GLUT4	Glucose transporter 4
WAT	White adipose tissue
HIF1a	Hypoxia-inducible factor 1a
LPL	Lipoprotein lipase
ATGL	Adipose triglyceride lipase
HSL	Hormone sensitive lipase
AMP	Adenosine monophosphate
ATP	Adenosine triphosphate
AMPK	Adenosine monophosphate-activated protein kinase
mTOR	Mammalian target of rapamycin
NAD+	Nicotinamide adenine dinucleotide
NADH	Reduced nicotinamide adenine dinucleotide
PARP-1	ADP-ribosyltransferase poly ADP-ribose polymerase 1
SIRT1	Sirtuin 1
HFD	High-fat diet
F6P	Fructose-6-phosphate
HBP	Hexosamine biosynthetic pathway
GFPT1	Glutamine-fructose-6-phosphate transaminase 1
O-GlcNAc	*O*-linked b-D-N-acetylglucosamine
SFA	Saturated fatty acid
IGF-1	Insulin-like growth factor-1
DMH	Dorsomedial hypothalamus
VMH	Ventromedial hypothalamus
PVN	Paraventricular nucleus
Arc	Arcuate nucleus
PUFA	Poly-unsaturated fatty acid
GHSR	Growth hormone secretagogue receptor
FEO	Food-entrainable oscillator
GLP-1	Glucagon-like peptide-1
AgRP	Agouti-related peptide
NPY	Neuropeptide Y
POMC	Pro-opiomelanocortin
CART	Cocaine- and amphetamine-regulated transcript
MC4R	Melanocortin 4 receptors
a-MSH	a-melanocyte-stimulating hormone
TRH	Thyrotropin-releasing hormone
TMN	Tuberomammillary nucleus
FAA	Food anticipatory activity
LH	Lateral hypothalamus
MCH	Melanin-concentrating hormone
